# Supplementary figures and images for: Streamlining N-terminally anchored yeast surface display via structural insights into S. cerevisiae Pir proteins
Source: Microb Cell Fact. 2023 Sep 7;22:174. doi: 10.1186/s12934-023-02183-2 (PMC10483737; doi:10.1186/s12934-023-02183-2)

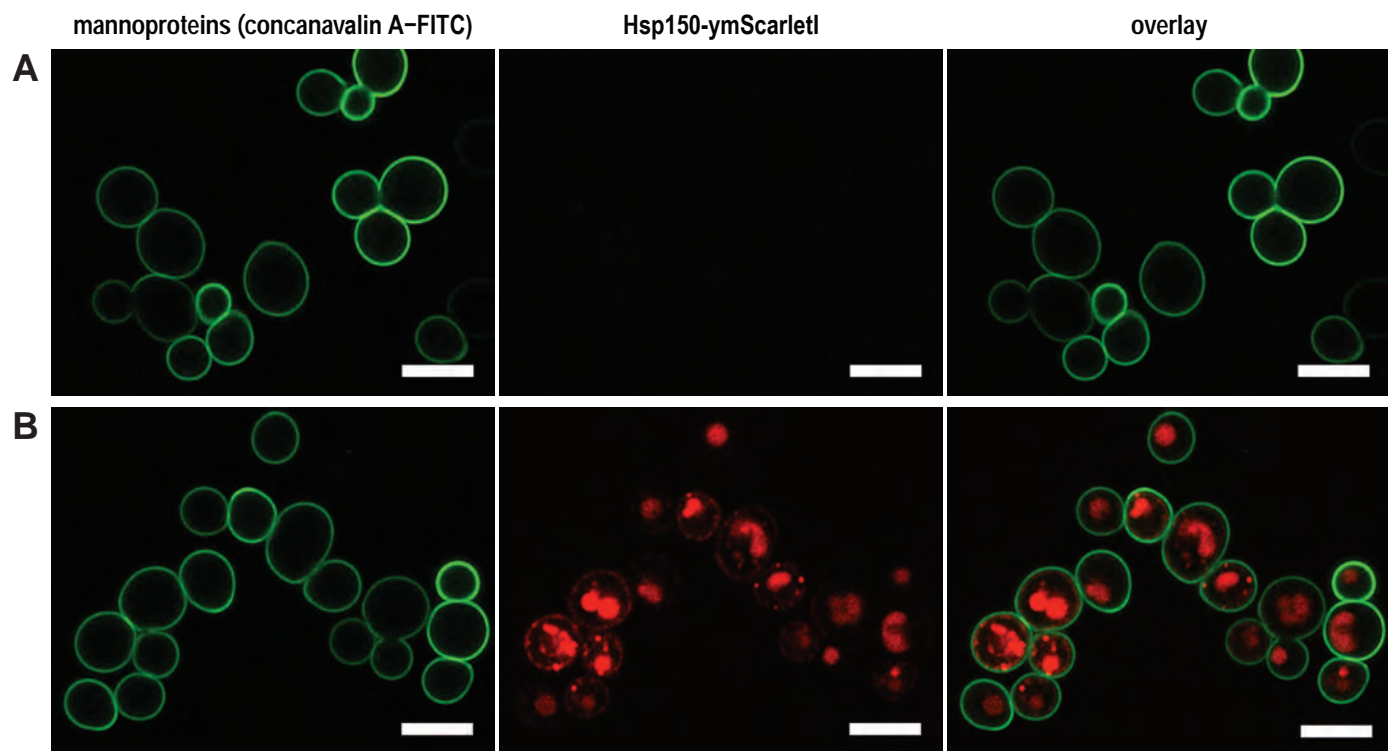

Supplementary Figure 1

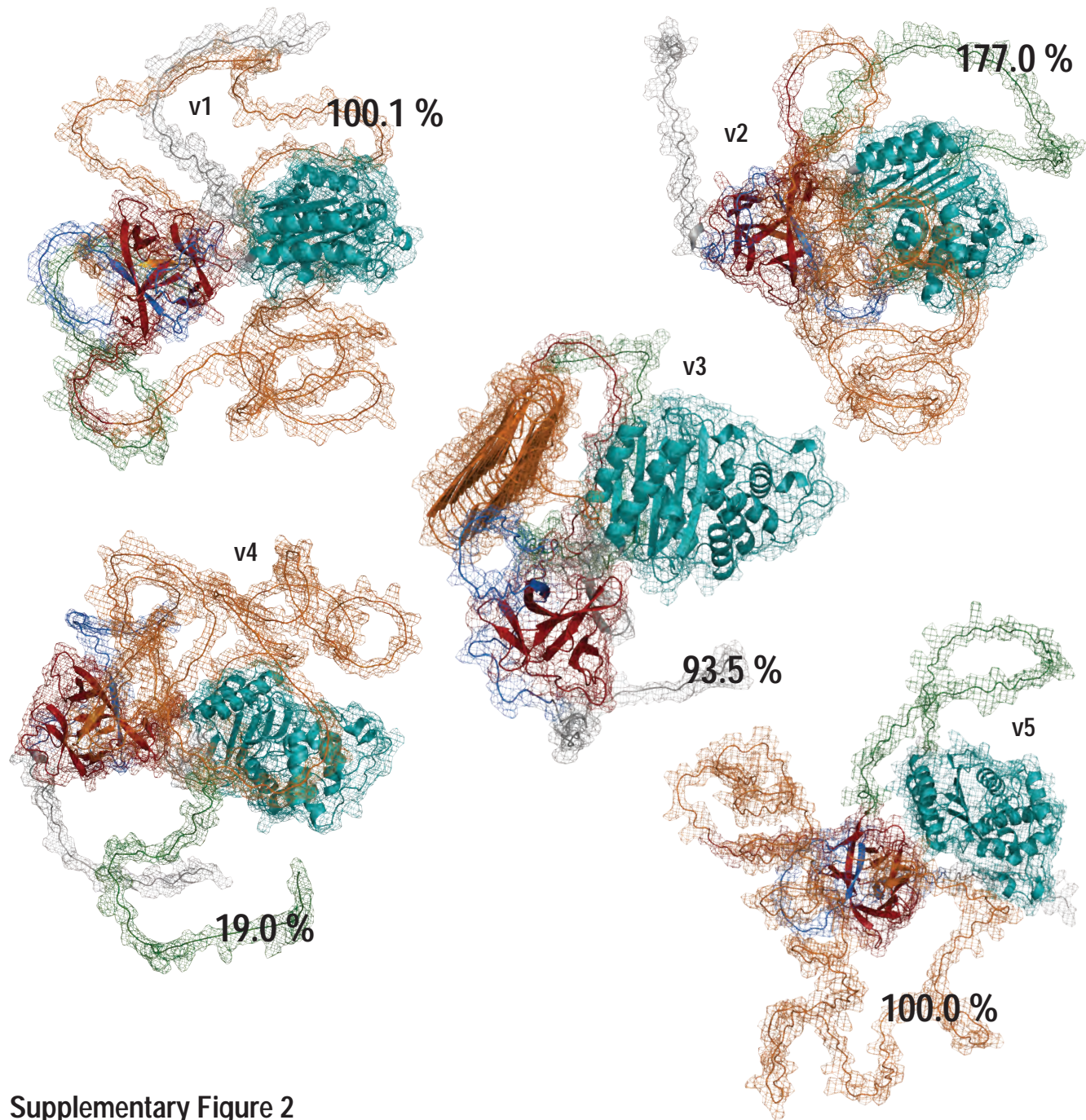

Supplementary Figure 2

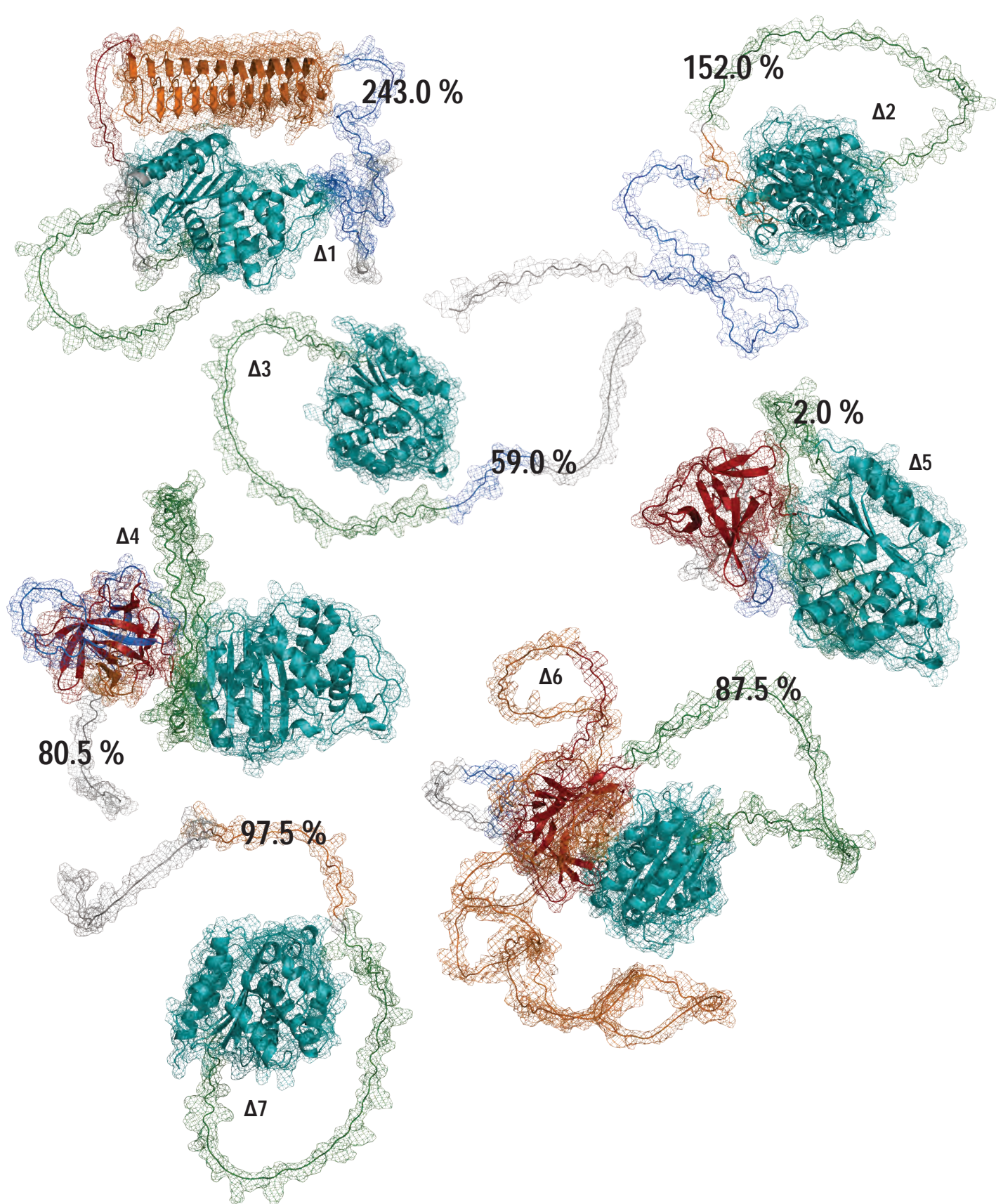

Supplementary Figure 3

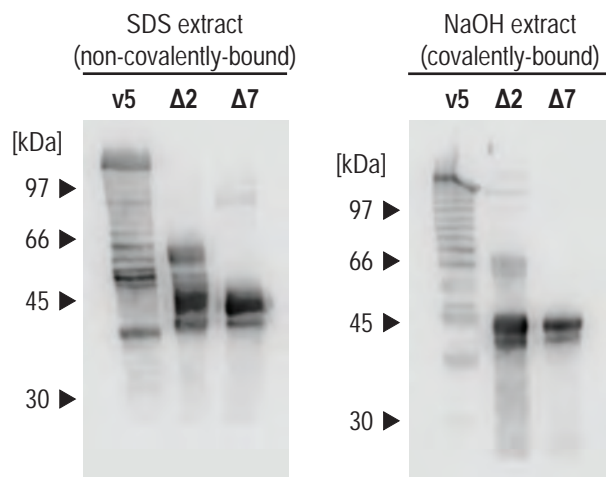

**Supplementary Figure 4**

Supplement: Supplementary file 2 — Supplementary Material 2: Supplementary Fig. 1 Single-channel micrographs and their overlays of (A) wild-type cells stained with FITC-conjugated concanavalin A and (B) Hsp150-ymScarletI-expressing cells stained with FITC-conjugated concanavalin A, as shown in Fig. 4. Scale bar denotes 5.0 μm. Fig. 2 Alphafold2-structural models of constructs v1-v5 of the Hsp150-β-lactamase fusion protein. Percentages denote the relative β-lactamase activity of the fusion construct, normalised to that of construct v5. The colour schemes of the structural models follow Fig. 5A. Fig. 3 Alphafold2-structural models of constructs Δ1-Δ7 of the Hsp150-β-lactamase fusion protein. Percentages denote the relative β-lactamase activity of the fusion construct, normalised to that of construct v5. The colour schemes of the structural models follow Fig. 6A. Supplementary Fig. 4 Comparison of the noncovalently- and covalently-bound cell wall proteins v5, Δ2, and Δ7, with anti-HA immunoblotting. [file 12934_2023_2183_MOESM2_ESM.pdf]
